# Resuscitation of Severe Accidental Hypothermia to Normal Neurologic Outcome With Use of Extracorporeal Membrane Oxygenation

**DOI:** 10.7759/cureus.15456

**Published:** 2021-06-05

**Authors:** Jamie Allen, Megan D Wardak, Rovinder S Sandhu, Ahmed R Nassar, Marna R Greenberg

**Affiliations:** 1 Emergency Medicine, Lehigh Valley Health Network, Allentown, USA; 2 General Surgery, Lehigh Valley Health Network, Allentown, USA; 3 Trauma Surgery, Lehigh Valley Health Network, Allentown, USA; 4 Cardiothoracic Surgery, Lehigh Valley Heart Institute, Allentown, USA; 5 Department of Emergency Medicine, Morsani College of Medicine/Lehigh Valley Health Network, Allentown, USA

**Keywords:** hypothermia, resuscitation, extracorporeal membrane oxygenation support, ecmo

## Abstract

Accidental hypothermia is a condition associated with significant morbidity and mortality. A 48-year-old male with a history of alcohol use disorder and optic neuropathy presented to the emergency department after being found unresponsive with an unknown downtime. One hundred four minutes passed from resuscitation, to pre-hospital discovery, until cannulation with extracorporeal membrane oxygenation. Here, a rare case of successful resuscitation of a profoundly hypothermic patient to normal neurologic outcome is presented.

## Introduction

Accidental hypothermia is a condition associated with significant morbidity and mortality with the worst outcomes in patients with initial core temperatures <24˚C [1]. Often these patients arrive at the emergency department (ED) in cardiac arrest despite pre-hospital resuscitation efforts. We present a case of successful resuscitation of a profoundly hypothermic patient to normal neurologic outcome who presented to an ED located in a temperate climate.

## Case presentation

A 48-year-old male with a history of alcohol use disorder and optic neuropathy presented to the ED after being found unresponsive on a sidewalk with an unknown downtime. Advanced cardiac life support (ACLS) was initiated for 40 minutes prior to ED arrival as the patient's initial rhythm fluctuated between fine ventricular fibrillation and asystole. His initial esophageal temperature was 21.1 ˚C.

The patient underwent rapid sequence intubation for airway protection. Additional peripheral venous access was obtained and fluid warmed to 40˚C was started. Bilateral chest tubes were placed and warmed fluid lavage began. Warm bladder irrigation was initiated. Initial serum laboratory values can be seen in Table 1. 

**Table 1:** Initial laboratory test results and normal ranges

	Patient results	Normal Adult Range
Blood urea nitrogen	41 mg/dL	7-28 mg/dL
Creatinine	2.33 mg/dL	0.53-1.30 mg/dL
Potassium	5.1 mmol/L	3.5-5.2 mmol/L
Bicarbonate	8 mEq/L	21 - 26 mEq/L
Phosphorus	4.9 mg/dL	2.3-4.6 mg/dL
Lactate	13.7 mmol/L	0.5-2.1 mmol/L

The Extracorporeal Membrane Oxygenation (ECMO) team was called just after patient arrival; while they assembled, an additional 45 minutes of ACLS was performed using standard protocols including epinephrine and defibrillation. Prior to cannulation and while undergoing mechanical CPR, the patient spontaneously opened his eyes. Asystole was noted at the subsequent pulse check. Upon restarting chest compressions, the patient again spontaneously opened his eyes and gave a “thumbs-up” when commanded. Consistently, if compressions were not provided, the patient was in asystole. Resuscitation techniques did not change as core temperature rose. The total duration of resuscitation, from pre-hospital discovery until cannulation with ECMO was 104 minutes.

Due to the geographic proximity in our hospital and surgeon preference, the patient was transported to the OR and underwent successful ECMO cannulation. He reached normal core temperature after approximately 3 hours but remained on veno-arterial ECMO and ventilatory support for 3 days due to cardiogenic shock that ultimately resolved. Initial Glasgow Coma Scale (GCS) was 3, but frequent neurologic checks showed a favorably improving exam.

The patient developed a small to moderate-sized hematoma at his right femoral access site that was evacuated without sequelae. Otherwise, he had an unremarkable remaining hospital course. He was discharged on hospital day 19 to a rehabilitation center with a normal neurologic physical exam (GCS 15), and organ function. Specifically, in the discharge summary, the patient reported “feeling well” and was participatory in conversation. His exam showed him to be alert and cooperative. While he was noted to have a flat affect, he was eager to go to rehab to recover.

## Discussion

Accidental hypothermia is an uncommon occurrence in most EDs. Survival is dependent upon multiple factors including degree of hypothermia, hypoxia prior to hypothermia, development of organ failure such as acute respiratory distress syndrome during hospital stay, elevated serum potassium, and others [2]. Treatment advancements, including a better understanding of peripheral warming, and the availability and growth of ECMO teams, have led to increased survival rates and favorable outcomes [1]. Despite these advancements, ECMO may not be routinely used in all EDs; cases such as ours that illustrate its advantages may continue to encourage clinicians’ interest in utilization.

Core temperature measurement upon presentation is an important first step as hypothermia staging will guide management. The Swiss Staging System of Hypothermia divides initial core temperatures into hypothermia (HT) stages. The ranges include: HT-1 (35˚C-32˚C), HT-2 (32˚C-28˚C), HT-3 (28˚C-24˚C), HT-4 (24˚C-13.7˚C), and HT-5 (<13.7˚C). Progression of cardiac instability and arrest is most commonly observed in patients in stage HT-4 or lower [3].

As stated above, rewarming efforts are guided by the hypothermia stage. Stage 1 patients can be rewarmed by passive measures such as warm blankets. In stage 2, treatment considerations include active external and minimally invasive rewarming techniques such as heating packs or blankets and/or warmed intravenous fluids. In patients with stage 3 hypothermia or worse, treatment is often dependent on hemodynamic stability, institutional capabilities, and available resources. Invasive measures such as peritoneal lavage or hemodialysis may be initiated on stable patients. Patients with one or more frozen limbs, severe hemodynamic instability, or cardiac arrest should undergo ECMO rewarming. Survival and survival to favorable neurologic outcomes have best been obtained with use of ECMO [4]. Additionally, the HOPE Score assists clinicians by predicting survival probability in hypothermic cardiac arrest patients undergoing ECMO rewarming [5].­ 

Due to lack of collateral information and circumstances surrounding our patient’s hypothermia, we opted to initiate both peripheral and invasive warming while simultaneously alerting cardiothoracic surgery about ECMO. His initial lab work was favorable (i.e. normal potassium level), and he was subsequently placed on ECMO support. While it is necessary to avoid using the neurological exam to influence the aggressiveness of the resuscitation and potential decision to continue the efforts, providers in this case were encouraged by the patient’s unique demonstration of ability to follow commands while receiving CPR. Our temperate-climate located ED infrequently treats profound accidental hypothermia and our eCPR program had yet to be implemented. This case underscores the importance that all ED providers should be prepared to initiate immediately available warming methods and ECMO-capable institutions should develop care pathways to treat profound hypothermia regardless of geographic location. The total duration of resuscitation, from pre-hospital discovery until cannulation with ECMO was 104 minutes.

## Conclusions

Accidental hypothermia is often associated with significant morbidity and mortality rates, and is an uncommon occurrence in most EDs. Over the years, advancements within treatment have been made, but due to lack of routine use within most EDs, physicians find it difficult to treat hypothermia. This case brings light to the importance that all ED physicians should be aware of available warming methods, specifically for when care needs to be initiated immediately. Emergency department clinicians should use our case as encouragement to utilize ECMOs and recognize its advantages. Lastly, ECMO-capable institutions should investigate developing care pathways to aid in treatment of hypothermia in all geographic locations.
